# Other PHAC publications

**DOI:** 10.24095/hpcdp.45.11/12.05

**Published:** 2025

**Researchers from the Public Health Agency of Canada also contribute to work published in other journals and books. Look for the following articles published in 2025:**

de Rubeis V, Wang E, Joshi D, Catherine N, MacMillan HL, Waddell C, […] **Tonmyr L**, et al. History of childhood maltreatment, prenatal cortisol levels, and executive functioning: a cross-sectional study using data from the Healthy Foundations Study. Child Abuse Neglect. 2025;167:107602. https://doi.org/10.1016/j.chiabu.2025.107602

**Prince SA, Manoharan N, Butler GP, Waites S, Shakeel N**. The influence of neighbourhood walkability and bikeability on park visits using mobility data in Canada. J Cycl Micromobil Res. 2025;5:100079. https://doi.org/10.1016/j.jcmr.2025.100079

Raza SZ, Whitten C, Summers A, **Randell S**, Denic N. Establishing a regional drug profile in Newfoundland and Labrador, Canada, using data from acute drug deaths. Clin Toxicol (Phila). 2025;63(9):640-7. https://doi.org/10.1080/15563650.2025.2529017

